# Clinical significance of miR-6515-5p in predicting diagnosis and prognosis for acute respiratory distress syndrome suffering from pulmonary fibrosis

**DOI:** 10.1186/s41065-025-00436-1

**Published:** 2025-04-26

**Authors:** Xueqin Huang, Peng Xu, Guangwen Long, Feihong Huang, Qian Zhang, Xiulin Yang, Hongpeng Sun, Chunling Ji, Wang Liu

**Affiliations:** 1 Department of Respiratory Medicine, 900th Hospital of the Joint Logistics Support Force of the Chinese People’s Liberation Army, Cangshan Campus, Fuzhou, 350007 Fujian Province China; 2 Department of Emergency, Wuhan Brain Hospital, General Hospital of the YANGTZE River Shipping, Wuhan, Hubei China; 3 Department of Emergency, Guizhou Provincial People’s Hospital, No. 83, Zhongshan East Road, Guiyang, 550002 Guizhou China; 4 Department of Integrated Section, Wushan Hospital Hangzhou First People’s Hospital (Hangzhou Cancer Hospital), No.34, Yanguan Lane, Zhongshan South Road, Shangcheng District, Hangzhou, 310002 Zhejiang Province China

**Keywords:** miR-6515-5p, ARDS, Pulmonary fibrosis, Diagnosis, Biomarker

## Abstract

**Background:**

The high mortality rate of ARDS is closely related to pulmonary fibrosis (PLF), and the degree of pulmonary fibrosis affects the prognosis of ARDS. Numerous studies indicated the abnormal expression of miRNAs in the pathogenesis of various lung diseases, such as ARDS and PLF. This study aimed to explore the expression of serum miR-6515-5p and its clinical performance in ARDS complicated with PLF.

**Methods:**

RT-qPCR analysis was employed to measure miR-6515-5p levels within the serum specimens from ARDS patients who either had PLF or did not. Receiver Operating Characteristics (ROC) curve was conducted to evaluate the diagnostic value of miR-6515-5p. To analyze the risk factors linked with the development of PLF in ARDS patients, logistic regression analysis was conducted. Kaplan-Meier curve was conducted to assess the prognostic value of miR-6515-5p in predicting the outcome of ARDS patients with PLF. Multivariate COX regression analysis was performed to identify the PLF-related risk factors associated with outcomes.

**Results:**

Serum miR-6515-5p was downregulated in ARDS patients, especially in patients suffering from PLF. miR-6515-5p expression can distinguish ARDS patients from healthy individuals and related to the occurrence of PLF. Most patients with low miR-6515-5p expression had low FVC and DLCO, as well as high Murray score and APACHE II score. Moreover, miR-6515-5p expression has a certain high value in differentiating ARDS patients with PLF from those without PLF. In addition, miR-6515-5p may be a prognostic marker in ARDS patients suffering from PLF.

**Conclusions:**

These data identified the abnormal expression of miR-6515-5p in ARDS and PLF, and implied a potential clinical early diagnostic and prognostic marker in ARDS suffering from PLF.

## Background

Acute respiratory distress syndrome (ARDS) is a life-threatening condition characterized by progressive dyspnea, acute onset of non-cardiogenic pulmonary edema, and severe hypoxemia [1]. As the disease progresses, one of the potential and dreaded complications is the development of pulmonary fibrosis (PLF). PLF is a process in which damaged lung tissue repairs itself and leaves scars. This abnormal fibrotic remodeling of the lung parenchyma severely impairs lung compliance and gas diffusion capacity [2]. The presence of pulmonary fibrosis in ARDS leads to a significantly worse prognosis [3]. Early diagnosis of ARDS and its potential progression to pulmonary fibrosis is of utmost importance as it could allow for prompt initiation of appropriate therapeutic interventions, such as lung-protective ventilation strategies [4]. Additionally, early identification of biomarkers or imaging features that can predict the progression of PLF in ARDS patients would enable personalized treatment approaches, potentially improving patient outcomes and reducing the long-term burden of this devastating disease.

microRNAs (miRNAs) represent a group of small, non-coding RNA molecules that assume crucial regulatory functions across various biological processes, including cell proliferation, differentiation, and inflammation [5, 6]. Recently, numerous studies have indicated the involvement of miRNAs in the pathogenesis of various lung diseases, such as ARDS and PLF [7, 8]. For instance, miR-346 levels were increased in lung tissues of ARDS and could regulate the development of ARDS by modulating the function of pulmonary microvascular endothelial cells [9]. Understanding the role of miRNAs in ARDS-related PLF could provide valuable insights into the disease mechanism and identify potential therapeutic targets [10]. Moreover, miRNAs may serve as potential biomarkers for the early diagnosis, prognosis, and prediction of disease progression in diseases, including ARDS [11–14]. Recently, an interaction network study has reported differentially expressed ncRNAs in idiopathic pulmonary fibrosis, in which miR-6515-5p was downregulated in idiopathic pulmonary fibrosis [15]. Although ARDS-related PLF and idiopathic pulmonary fibrosis have some differences in etiology, they share common pathological features in the development of fibrotic lesions, such as fibroblast activation and excessive extracellular matrix deposition. Another study indicated that miR-6515-5p was downregulated in PM10-exposed human bronchial epithelial BEAS-2B cells and could regulate PM10-induced inflammatory responses by targeting CSF3 via MAPK/ERK signaling in BEAS-2B cells [16]. Given the emerging role of miRNAs as key regulators in pulmonary fibrosis and the fact that miR-6515-5p shows abnormal expression in idiopathic pulmonary fibrosis, it is reasonable to hypothesize that miR-6515-5p may also play a significant role in ARDS-related PLF.

Previous research on the pathogenesis of ARDS complicated by PLF has primarily centered on traditional molecular pathways and protein-based biomarkers. However, our study uniquely zeroes in on miR − 6515–5p, a microRNA that has hitherto received scant attention in this context. Understanding the role of miR-6515-5p in ARDS-PLF might provide a promising biomarker for diagnosis, prognosis, and prediction of disease progression in ARDS-PLF patients. In this research, our objective was to explore the expression pattern and clinical value of miR-6515-5p in ARDS patients with and without PLF. These findings may potentially improve our understanding of the pathogenesis of ARDS-related PLF and serve as a stepping-stone for the formulation of miR-6515-5p-based diagnostic and therapeutic strategies.

## Methods

### Study participants

A total of 210 ARDS patients admitted to Guizhou Provincial People’s Hospital from 2022 to 2024 were selected as the study subjects. The inclusion criteria were: (1) all cases met the Berlin criteria or the new criteria ESICM guidelines [17, 18]; (2) with less than 7 days of disease course; (3) patients completed follow-up records and with complete clinical data; 3) all patients signed written informed consent. The following cases were not included: (1) those with malignant tumors; (2) cases had a history of lesions in important organs, such as the liver and kidneys; (3) those suffering from immune system diseases. All enrolled subjects were subjected to chest high-resolution computed tomography (HRCT) to assess lung fibroproliferation. Then, the HRCT findings were then utilized to classify participants into two groups, including 121 ARDS patients without PLF and 89 patients with PLF. Additionally, 88 healthy individuals undergoing routine physical examinations in the same time frame were enrolled as the health control group. Inclusion criteria for the control group included: (1) absence of any respiratory symptoms or history of lung disease; (2) normal chest radiography findings; (3) written informed consent obtained.

This study was approved by the Medical Ethical Committee of Guizhou Provincial People’s Hospital. The clinical data were collected and recorded for further analysis.

### Sample collection

Fasting peripheral venous blood samples were collected from patients within 24 h of diagnosis and from healthy controls during routine physical examinations. All samples were centrifuged at 1000 g for 10 min at 4 °C. The supernatant serum was aspirated and aliquoted into 1.5 mL cryovials (Eppendorf, Hamburg, Germany) and stored at -80 °C until analysis, with no more than 3 freeze-thaw cycles.

### RNA isolation and real-time quantitative polymerase chain reaction (RT-qPCR)[19, 20]

The expression of miR-6515-5p was detected by real-time quantitative polymerase chain reaction (RT-qPCR). Total RNA was extracted from serum samples utilizing Trizol reagent (Invitrogen). For each serum sample, 500 µL of Trizol reagent was added to the serum in a 1.5 - mL microcentrifuge tube. The tube was then vortexed vigorously for 15 s to ensure thorough mixing and incubated at room temperature for 5 min to allow for the complete dissociation of nucleoprotein complexes. Next, 100 µL of chloroform was added to the tube, which was then vortexed again for 15 s and incubated at room temperature for another 3 min. After incubation, the samples were centrifuged at 12,000 g for 15 min at 4 °C. The upper aqueous phase containing the RNA was carefully transferred to a new RNase - free microcentrifuge tube, taking care not to disturb the interphase. To precipitate the RNA, an equal volume of isopropanol was added to the aqueous phase, mixed gently by inversion, and incubated at room temperature for 10 min. The samples were then centrifuged at 12,000 g for 10 min at 4 °C. The supernatant was discarded, and the RNA pellet was washed with 75% ethanol (prepared with RNase - free water). The tube was centrifuged at 7,500 g for 5 min at 4 °C, and the ethanol supernatant was carefully removed. The RNA pellet was air-dried for 5–10 min and then resuspended in 30 µL of RNase-free water. To minimize experimental errors during RNA extraction, all glassware and plasticware used were pre-treated with RNase-decontamination solutions and autoclaved. Pipette tips were RNase-free and filter-tipped to prevent cross-contamination. All steps were carried out as quickly as possible to reduce the risk of RNA degradation. The concentration and purity of the extracted RNA were measured by a NanoDrop spectrophotometer (Thermo Fisher). Then the extracted RNA was reverse-transcribed into cDNA using the TaqMan MicroRNA Reverse Transcription Kit (Applied Biosystems). The PCR was performed using the TaqMan Universal Master Mix II (Applied Biosystems) on a StepOnePlus Real-Time PCR System (Applied Biosystem). The thermal cycle was run in the following program: 95 °C for 10 min, 35 cycles of 95 °C for 15 s, and 58 °C for 45 s. The relative expression of miR-6515-5p was calculated using the 2^−ΔΔCt^ methods with U6 as the internal reference gene.

### Statistical analysis

All statistical analyses were performed using SPSS 26.0 (IBM, Armonk, NY, USA) and GraphPad Prism 9.0 (GraphPad Software, San Diego, CA). A two-tailed *P*-value < 0.05 was considered statistically significant. For normally distributed continuous variables, a Student t-test was employed to compare the differences between different groups. Categorical variables were compared using the chi-square test. The receiver operating characteristics (ROC) curve was constructed to evaluate the diagnostic performance of miR-6515-5p by calculating the area under the curve (AUC), sensitivity, and specificity. Logistic regression analysis was performed to explore risk factors associated with PLF. Kaplan-Meier curve, a commonly used method in survival analysi, was conducted to the prognostic value of miR-6515-5p in ARDS patients with PLF. A significant *P*-value suggests that the difference in miR-6515-5p levels is unlikely to be due to chance and may be clinically relevant for predicting prognosis.

## Results

### The baseline data of the participants

The healthy control group consisted of 88 individuals (mean age: 53.57 ± 7.13 years; 47 males, 41 females) with a mean body mass index (BMI) of 23.56 ± 3.71 kg/m², among whom 47 (53.4%) were smokers. Among these controls, 47 individuals reported a history of smoking. A total of 210 ARDS patients (mean age: 52.87 ± 8.23 years; 101 males, 109 females) were enrolled, with a mean body mass index (BMI) of 24.64 ± 3.61 kg/m² and 116 (55.2%) reporting a smoking history. No statistically significant differences were observed between groups in age (*P* = 0.489), sex (*P* = 0.403), BMI (*P* = 0.090), or smoking prevalence (*P* = 0.772), confirming baseline comparability (Table 1).

**Table 1:** Baseline data of subjects

characteristics	Healthy controls (*n* = 88)	ARDS patients (*n* = 210)	*P*-value
Age (years)	53.57 ± 7.13	52.87 ± 8.23	0.489
Sex (male/female)	47/41	101/109	0.403
BMI (kg/m^2^)	23.56 ± 3.71	24.64 ± 3.61	0.090
Smoking (no/yes)	41/47	94/116	0.772
WBC (×10^9^/L)	-	13.19 ± 4.39	-
CRP (mg/L)	-	50.86 ± 25.16	-
PCT (ng/mL)	-	1.08 ± 0.73	-
TNF-α (pg/mL)	-	24.09 ± 12.87	-
IL-1β (pg/mL)	-	20.62 ± 7.32	-
IL-6 (pg/mL)	-	98.83 ± 58.85	-
FVC (%)	-	81.19 ± 13.94	-
DLCO (%)	-	62.01 ± 14.08	-
Murray score	-	2.35 ± 1.07	-
APACHE II score	-	15.97 ± 5.99	-

Note: ARDS, acute respiratory distress syndrome; PLF, pulmonary fibrosis; WBC, white blood cells; CRP, c-reactive protein; PCT, procalcitonin; TNF-α, tumor necrosis factor-α; IL, interleukin; FVC, forced vital capacity; DLCO, diffusion lung CO; -, no data

### miR-6515-5p expression and its predictive value in distinguishing ARDS patients from healthy individuals

**Fig. 1:**
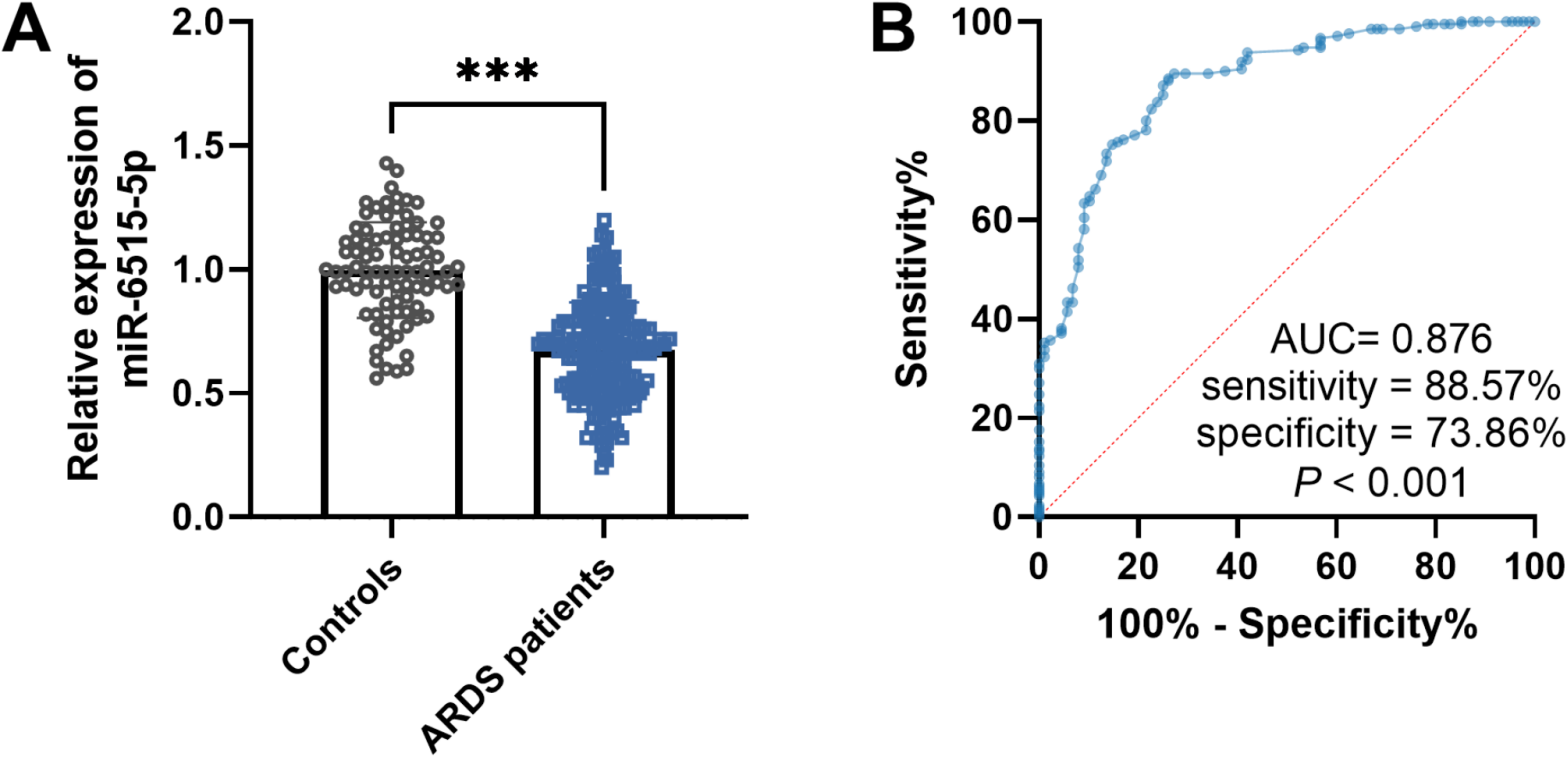
Serum miR-6515-5p expression and its clinical value in ARDS patients. ***P < 0.001. (**A**) Serum miR-6515-5p expression was decreased in ARDS patients compared to healthy control. (**B**) ROC curve was conducted to evaluate the diagnostic value of miR-6515-5p in distinguishing ARDS patients from healthy individuals

The serum miR-6515-5p levels were decreased in patients with ARDS in contrast to those in healthy individuals (Fig. 1A). The ROC curve indicated that miR-6515-5p expression had a relatively high area under the ROC curve (AUC) value in distinguishing ARDS patients from healthy individuals with a sensitivity of 88.57% and specificity of 73.86% (Fig. 1B).

### miR-6515-5p expression was associated with the severity of ARDS in patients

**Fig. 2:**
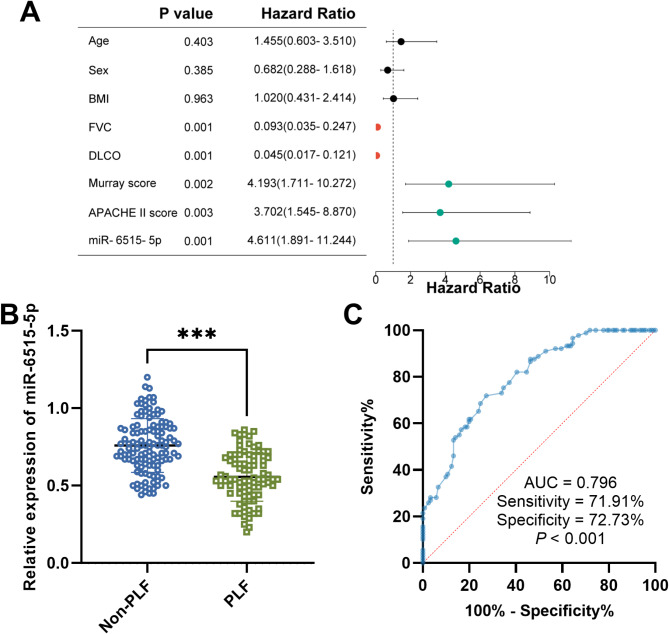
miR-6515-5p expression was a risk factor associated with the occurrence of PLF in ARDS patients. ***P < 0.001. (**A**) Logistic regression analysis was performed to analyze the risk factors in ARDS patients suffering from PLF. (**B**) miR-6515-5p levels were lower in ARDS patients with PLF than in patients without PLF. (**C**) miR-6515-5p has a certain diagnostic value in differentiating ARDS patients with PLF from those without PLF

Among the 210 ARDS patients, 89 cases suffered PLF. Firstly, the baseline data were compared between non-PLF groups and PLF groups in ARDS patients (Table 2). There were no statistical differences in age, sex, BMI, and smoking. The ARDS patients who suffered from PLF showed higher WBC, CRP, PCT, TNF-α, IL-6, Murray score, and APACHE II score, as well as lower FVC and DLCO values. Additionally, based on the median value of miR-6515-5p levels (0.67) in ARDS patients, the ARDS patients were classified into low miR-6515-5p expression group (*n* = 106) and high miR-6515-5p expression group (*n* = 104). The patients with low miR-6515-5p expression displayed lower FVC and DLCO, along with a higher Murray score, APACHE II score, and high incidence of PLF (Table 3). Then these clinical characteristics in Table 3 were enrolled in logistic regression analysis. The results in Fig. 2A indicated that FVC, DLCO, Murray score, APACHE II score, and miR-6515-5p expression were risk factors associated with the occurrence of PLF.

**Table 2:** Comparison of clinical characteristics between non-PLF and PLF groups in ARDS patients

Indicators	ARDS patients		*P* value
	Non-PLF (*n* = 121)	PLF (*n* = 89)	
Age (years)	52.60 ± 7.90	53.24 ± 8.71	0.583
Sex (male/female)	58/63	43/46	0.956
BMI (kg/m^2^)	24.23 ± 3.73	24.44 ± 3.18	0.658
Smoking (no/yes)	54/67	40/49	0.964
WBC(×10^9^/L)	12.31 ± 4.13	14.38 ± 4.48	< 0.001
CRP (mg/L)	36.43 ± 18.04	70.48 ± 19.59	< 0.001
PCT (ng/mL)	0.99 ± 0.64	1.19 ± 0.82	0.059
TNF-α (pg/mL)	20.31 ± 12.80	29.23 ± 11.13	< 0.001
IL-1β (pg/mL)	21.21 ± 8.17	19.82 ± 5.92	0.173
IL-6 (pg/mL)	81.53 ± 51.39	122.40 ± 60.44	< 0.001
FVC (%)	87.02 ± 9.70	73.28 ± 14.94	< 0.001
DLCO (%)	70.70 ± 6.52	50.20 ± 12.95	< 0.001
Murray score	2.22 ± 1.09	2.54 ± 1.02	0.030
APACHE II score	14.98 ± 6.22	17.33 ± 5.42	0.005

Note: ARDS, acute respiratory distress syndrome; PLF, pulmonary fibrosis; WBC, white blood cells; CRP, c-reactive protein; PCT, procalcitonin; TNF-α, tumor necrosis factor-α; IL, interleukin; FVC, forced vital capacity; DLCO, diffusion lung CO

**Table 3:** Association between miR-6515-5p expression and clinical features of ARDS patients

	Total (*n* = 210)	Low miR-6515-5p expression (*n* = 106)	High miR-6515-5p expression (*n* = 104)	*P*-value
Age (years)				0.400
< 55	113	54	59	
≥ 55	97	52	45	
Sex				0.404
Male	101	54	47	
Female	109	52	57	
BMI (kg/m^2^)				0.680
≤ 24	104	51	53	
> 24	106	55	51	
FVC (%)				0.034
< 81	88	52	36	
≥ 81	122	54	68	
DLCO (%)				0.015
< 62	82	50	32	
≥ 62	128	56	72	
Murray score				0.019
≤ 2.5	102	43	59	
> 2.5	108	63	45	
APACHE II score				0.013
≤ 15	109	46	63	
> 15	101	60	41	
PLF				< 0.001
No	121	43	78	
Yes	89	63	26	

Note: ARDS, acute respiratory distress syndrome; PLF, pulmonary fibrosis; FVC, forced vital capacity; DLCO, diffusion lung CO

### miR-6515-5p expression and its clinical significance in ARDS patients suffering from PLF

The miR-6515-5p expression was further compared in ARDS patients without PLF and with PLF. Patients with PLF had lower miR-6515-5p levels (Fig. 2B). ROC curve analysis revealed that miR-6515-5p expression had a relatively high AUC value (0.796) to differentiate PLF from non-PLF in ARDS patients (Fig. 2C).

In reference to the median value of miR-6515-5p expression in ARDS complicated with PLF patients, the cases were divided into two groups: low expression (*n* = 47) and high expression (*n* = 42) groups. Consistent with that in all ARDS patients, most patients with low miR-6515-5p expression had low FVC and DLCO, as well as high Murray score and APACHE II score (Table 4).

**Table 4:** Association between miR-6515-5p expression and clinical features of ARDS complicated with pulmonary fibrosis patients

	Total (*n* = 210)	Low miR-6515-5p expression (*n* = 47)	High miR-6515-5p expression (*n* = 42)	*P*-value
Age (years)				0.110
< 55	45	20	25	
≥ 55	44	27	17	
Sex				0.176
Male	42	19	23	
Female	47	28	19	
BMI (kg/m^2^)				0.727
≤ 24	42	23	19	
> 24	47	24	23	
FVC (%)				0.025
< 73	43	28	15	
≥ 73	46	19	27	
DLCO (%)				0.021
< 50	39	26	13	
≥ 50	50	21	29	
Murray score				0.030
≤ 2.5	36	14	22	
> 2.5	53	33	20	
APACHE II score				0.029
≤ 15	40	16	24	
> 15	49	31	18	

Note: ARDS, acute respiratory distress syndrome; FVC, forced vital capacity; DLCO, diffusion lung CO

### The prognostic value of miR-6515-5p in ARDS patients with PLF

**Fig. 3:**
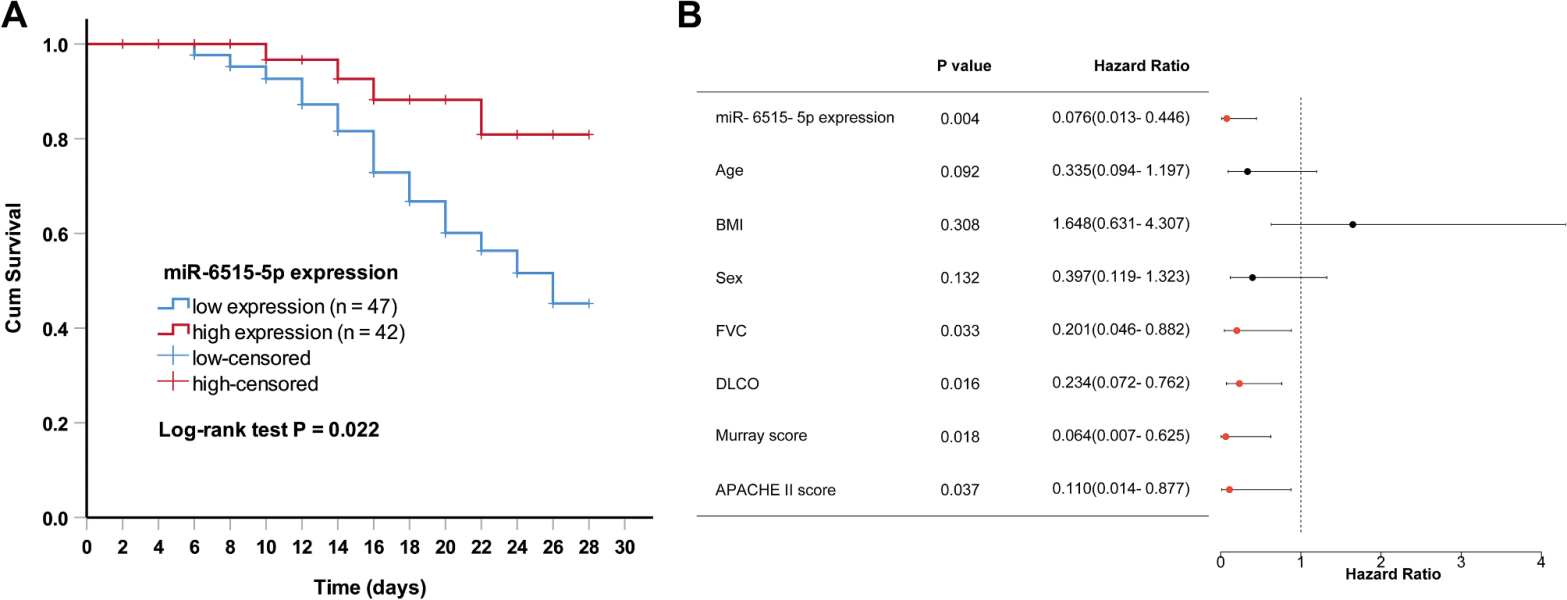
The prognostic value of miR-6515-5p in predicting outcomes in ARDS patients with PLF. (**A**) Kaplan-Meier curve was conducted to evaluate the prognostic value of miR-6515-5p. (**B**) Multivariate COX regression analysis was performed to analyze the independent risk factors associated with the outcome of ARDS patients with PLF

The miR-6515-5p expression and the 28-day follow-up data of the ARDS patients with PLF were enrolled in the Kaplan-Meier curve analysis. The results in Fig. 3A showed that patients with low miR-6515-5p had a lower survival rate (log-rank test *P* = 0.022). The multivariate COX regression analysis showed that miR-6516-5p expression was an independent prognostic factor associated with the patients’ outcomes (Fig. 3B).

## Discussion

An increasing number of treatment strategies are being continuously applied to the clinical treatment of various diseases in the medical field [21–23]. MiRNA-based therapy approaches were also applied to clinical studies in diseases [5, 24]. Previous investigations into the relationship between ARDS and PLF have largely focused on established cytokines, growth factors, and extracellular matrix components. In contrast, our study pioneers the exploration of miR-6515-5p as a key player in this complex disease network. We demonstrated for the first time that miR-6515-5p levels are not only significantly downregulated in ARDS patients, but also exhibit a strong correlation with the presence and severity of PLF. This novel finding expands the current molecular landscape of ARDS - PLF research, opening up new avenues for future therapeutic interventions. This study confirmed that serum miR-6515-5p expression was decreased in ARDS patients, especially in those suffering from PLF. miRNAs are known to play crucial regulatory roles in various biological processes, and their dysregulation has been implicated in numerous diseases [6]. The low expression of miR-6515-5p in ARDS patients may suggest its potential involvement in the pathogenesis of ARDS. In the context of ARDS with PLF, the even more pronounced downregulation implies that miR-6515-5p could be closely associated with the fibrotic process. Aberrant miRNA expression can disrupt normal cell functions, such as proliferation, differentiation, and apoptosis, which are processes that are dysregulated in ARDS and PLF [25–27]. The ARDS patients who suffered from PLF showed higher WBC, CRP, PCT, TNF-α, IL-6, Murray score, and APACHE II score, as well as lower FVC and DLCO values. These data showed ARDS patients had abnormal inflammatory factors and clinical evaluation scores. The patients with low miR-6515-5p expression displayed lower FVC and DLCO, along with a higher Murray score, APACHE II score, and high incidence of PLF. This further confirmed that abnormal miR-6515-5p expression was closely related to the disease progression of ARDS, especially the occurrence of PLF.

Circulating miRNAs are highly stable in body fluids and are considered promising biomarkers for disease diagnosis and therapy [24]. For instance, miR-338-3p is a biomarker related to the diagnosis and severity of neonatal ARDS and has a regulatory influence on inflammatory response in ARDS [28]. The expression of miR-6515-5p could effectively distinguish ARDS patients from healthy individuals, as well as ARDS patients with PLF from those without. This finding highlights the potential of miR-6515-5p as a diagnostic biomarker for early prediction of the occurrence of PLF in ARDS patients. In clinical practice, early and accurate diagnosis of ARDS and its subtypes is of utmost importance for timely intervention and improving patients’ outcomes [29]. Current diagnostic methods for ARDS often rely on clinical symptoms, imaging, and pulmonary function tests, which may lack specificity or sensitivity in the early stages. The identification of miR-6515-5p as a diagnostic biomarker provides a new perspective and may complement existing diagnostic tools, enabling more precise diagnosis and classification of ARDS patients.

PLF is a severe complication of ARDS, significantly increasing the morbidity and mortality of patients. Understanding the factors contributing to the development of PLF in ARDS is crucial for the development of preventive and therapeutic strategies. The logistic analysis indicated that the expression of miR-6515-5p was a risk factor for the development of PLF in ARDS patients. Previous study also indicated miRNA expression (miR-141-3p) was associated with the severe progression of PLF and was a risk factor for PLF in ARDS [30]. miR-6515-5p expression may influence the fibrotic process through various molecular mechanisms. For instance, miR-23b-3p alleviates endothelial-to-mesenchymal transition in PLF by suppressing the DPP4 expression [31]. A previous study in lung cancer demonstrated that miR-6515-5p dysregulation might facilitate lung cancer proliferation and metastasis [32]. miR-6515-5p within exosomes isolated from bone marrow mesenchymal stem cells plays a crucial role in exerting a protective effect on osteoarthritis, which occurs through a regulatory axis involving lncRNA LYRM4-AS1 and GRPR [33]. Downregulation of miR-6515-5p in PM10-exposed human bronchial epithelial BEAS-2B cells could regulate PM10-induced inflammatory responses by targeting CSF3 through MAPK/ERK pathway [16]. Given the previous study in lung epithelial cells and lung cancer, as well as the results in this study, we speculated that miR-6515-5p might involved in the progression of PLF by modulating CSF3 expression. Nevertheless, the functions and potential mechanism of miR-6515-5p in PLF remain unclear.

MiR-6515-5p was reported to be upregulated in small-cell lung cancer but has not been validated prognostic marker in lung cancer [34]. This study showed that miR-6515-5p expression might act as a prognostic biomarker for ARDS patients with PLF. The ARDS patients suffering from PLF with low miR-6515-5p levels displayed a lower survival rate. Prognosis prediction is an essential aspect of patient management, allowing clinicians to stratify patients according to their risk of adverse outcomes and design personalized treatment plans [18]. Lower levels of miR-6515-5p may be associated with a more severe disease course and worse outcomes in ARDS with PLF patients, which might be related to its role in promoting disease-related processes such as fibrosis and inflammation.

Despite these findings, our study has several limitations. First, the sample size in each group, especially in the subgroup analysis, may not be large enough to fully represent the entire patient population, which may restrict the broad applicability of our results. It is necessary to conduct future studies involving larger sample sizes to confirm our findings. Second, the study was mainly observational, underlying the role of miR-6515-5p in ARDS and its associated PLF remains to be further explored. Additionally, in-depth experiments will be conducted to identify the potential molecular mechanism of miR-6515-5p for ARDS with PLF.

## Conclusions

In conclusion, our study has identified miR-6515-5p as a potential biomarker for predicting diagnosis, risk-predicting, and prognosis values in ARDS and its associated PLF. Further research in this area may lead to significant advancements in the management of ARDS patients. To fully understand the underlying mechanisms by which miR-6515-5p exerts its effects, future research should focus on in-depth mechanistic studies. Additionally, larger-scale clinical validations are needed to confirm the reliability and generalizability of miR-6515-5p as a biomarker across diverse patient populations.

## Data Availability

All data generated or analyzed during this study are included in this article. Further enquiries can be directed to the corresponding author.

## Abbreviations

PLF: Pulmonary fibrosis
ROC: Receiver Operating Characteristics
ARDS: Acute respiratory distress syndrome
miRNAs: microRNAs
RT-qPCR: Real-time quantitative polymerase chain reaction
AUC: Area under the curve
BMI: Body mass index
